# Supplementary material for: Efficient and Stable Fiber Dye-Sensitized Solar Cells Based on Solid-State Li-TFSI Electrolytes with 4-Oxo-TEMPO Derivatives
Source: Nanomaterials (Basel). 2022 Jul 5;12(13):2309. doi: 10.3390/nano12132309 (PMC9267960; doi:10.3390/nano12132309)
Supplement: Supplementary file 1 [file nanomaterials-12-02309-s001.zip › nanomaterials-1784828-SI.pdf]

nanomaterials

Supplementary Materials

Efficient and Stable Fiber Dye-Sensitized Solar Cells Based on Solid-State Li-TFSI Electrolytes with 4-Oxo-TEMPO Derivatives

Pyeongje An [1,2,†], Jae Ho Kim [1,†], Myeonghwan Shin [3,†], Sukyeong Kim [1], Sungok Cho [1], Chaehyun Park [1,2], Geonguk Kim [1,2], Hyung Woo Lee [2,4,*], Jin Woo Choi [1,*], Chuljin Ahn [3,*] and Myungkwan Song [1,*]

[1] Department of Energy & Electronic Materials, Korea Institute of Materials Science (KIMS), Changwon 51508, Gyeongsangnam-do, Korea; apj6669@kims.re.kr (P.A.); jho83@kims.re.kr (J.H.K.); chae119@kims.re.kr (S.K.); choso1225@kims.re.kr (S.C.); tlsaudghks98@naver.com (C.P.); rndndndnr2@kims.re.kr (G.K.)
[2] Department of Nano Fusion Technology, Pusan National University, Busan 46241, Korea
[3] Department of Biology and Chemistry, Changwon National University, Changwon 51140, Gyeongsangnam-do, Korea; a463489@kims.re.kr
[4] Department of Nanoenergy Engineering and Research Center of Energy Convergence Technology, Pusan National University, Busan 46241, Korea
* Correspondence: lhw2010@pusan.ac.kr (H.W.L.); jinwoo.choi@kims.re.kr (J.W.C.); cjahn@changwon.ac.kr (C.A.); smk1017@kims.re.kr (M.S.)
† These authors contributed equally to this work.

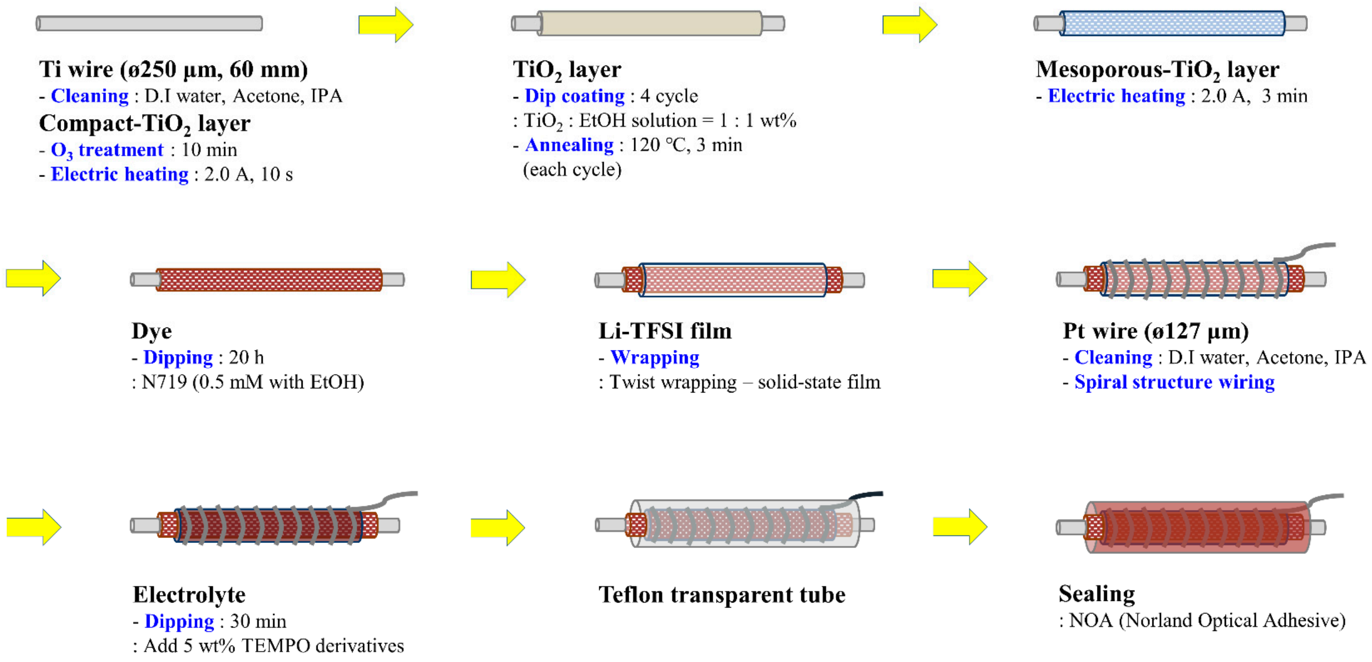

Figure S1. Fabrication process of TEMPO based SS-FDSSCs.

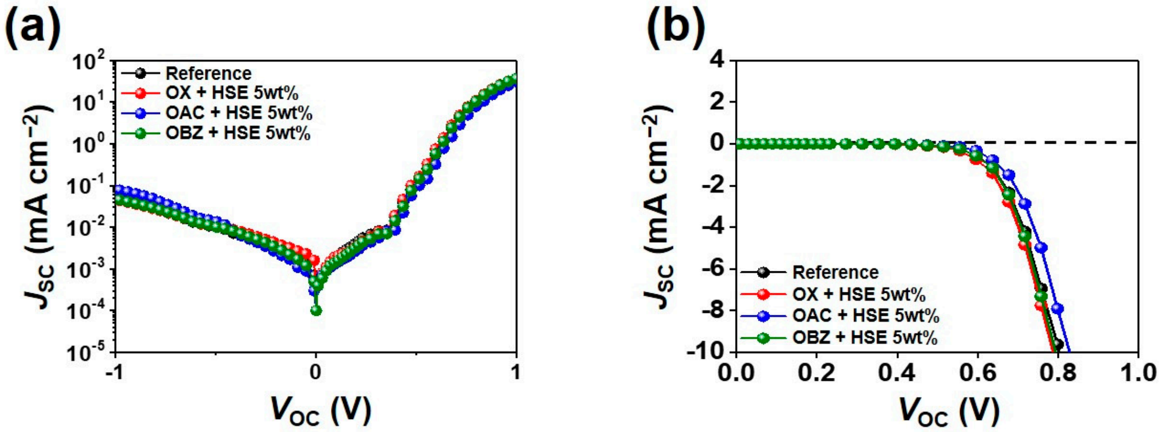

Figure S2. *J-V* characteristics of TEMPO-based SS-FDSSCs in the dark condition: (**a**) typical semi-logarithmic scale from −1 V to +1 V and (**b**) normal scale.

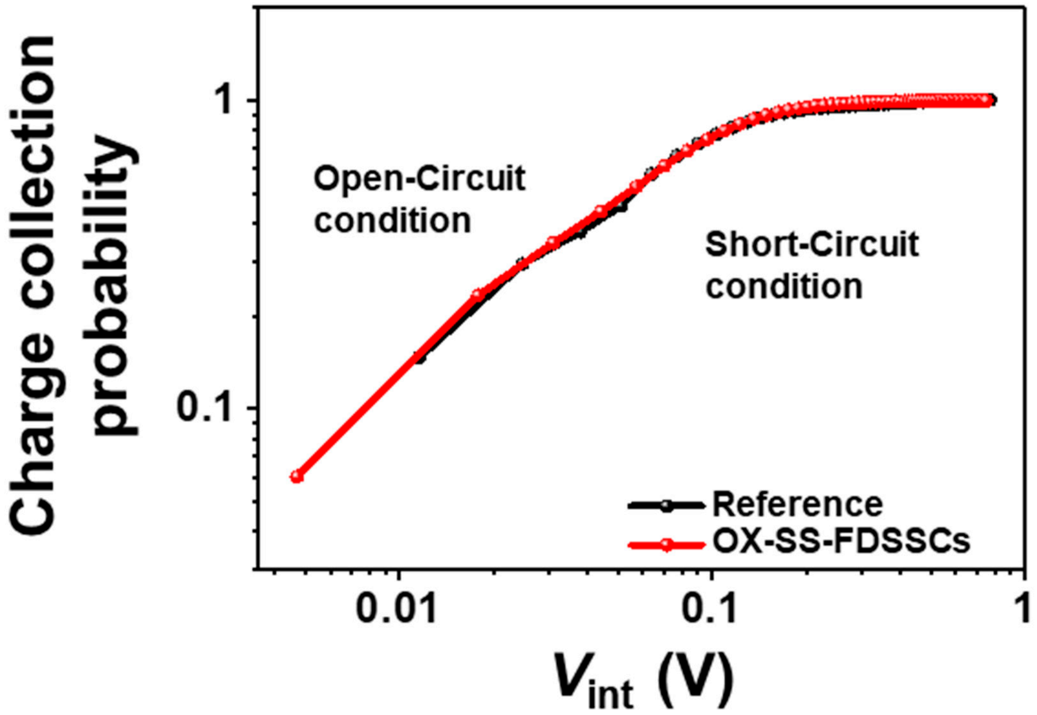

Figure S3. Characterization and photovoltaic properties of TEMPO-SS-FDSSCs devices: charge collection probability (or) normalized photocurrent with saturated photocurrent as a function of internal voltage.

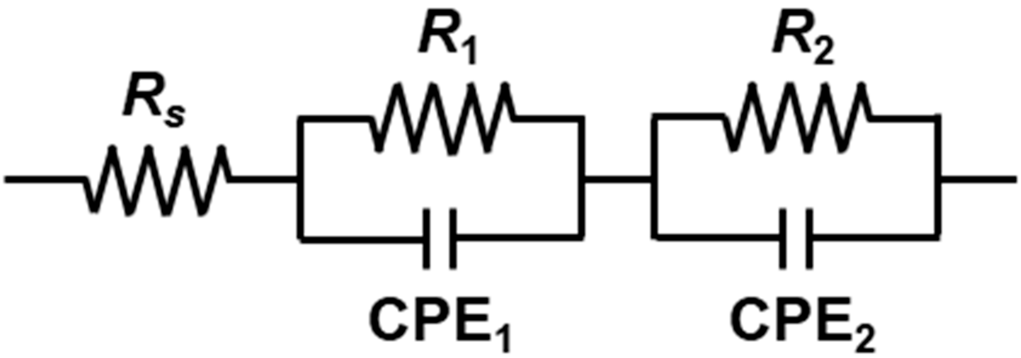

Figure S4. The equivalent circuit for EIS analysis.

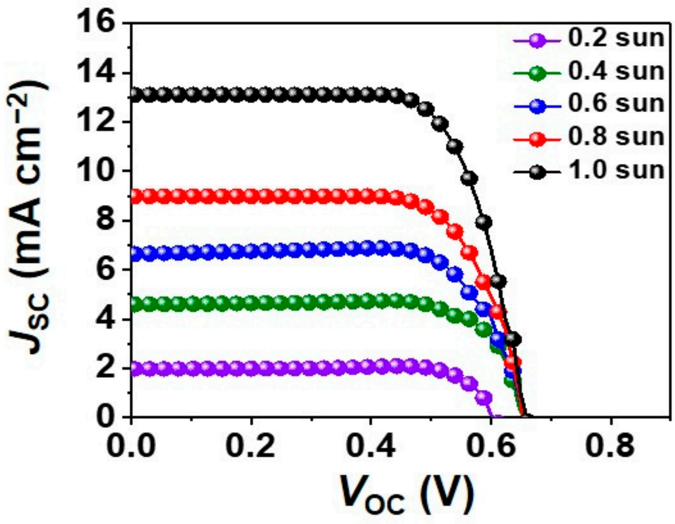

Figure S5. The *J*–*V* curves of the OX enhanced SS-FDSSC under various illuminations of 0.2 to 1.0 sun.

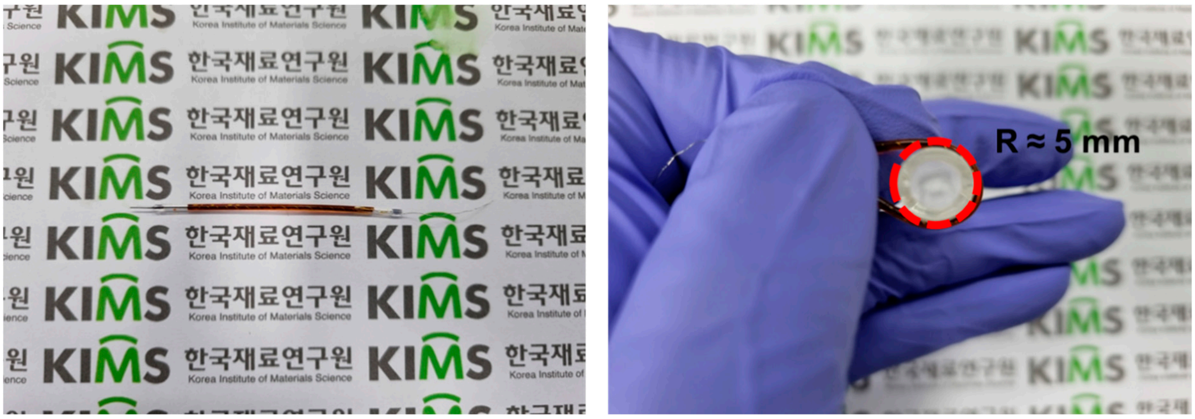

Figure S6. Photographs showing bending test for the OX-enhanced SS-FDSSC.

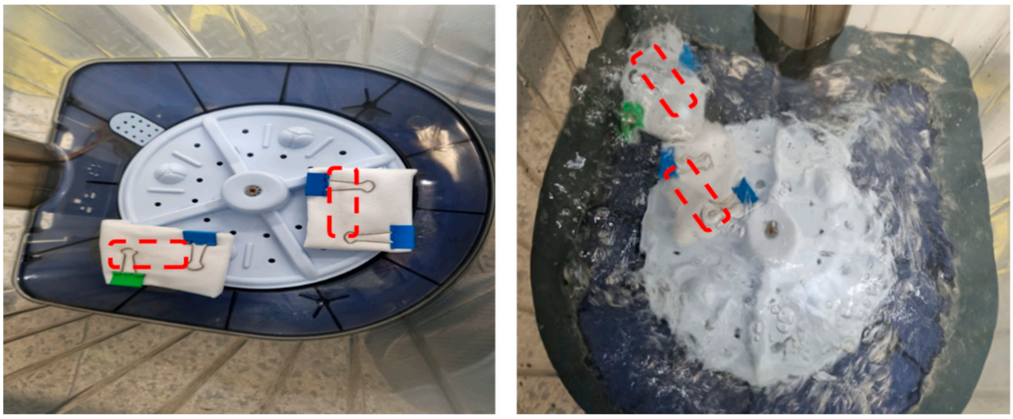

Figure S7. Photographs showing washing test for the OX-enhanced SS-FDSSC.

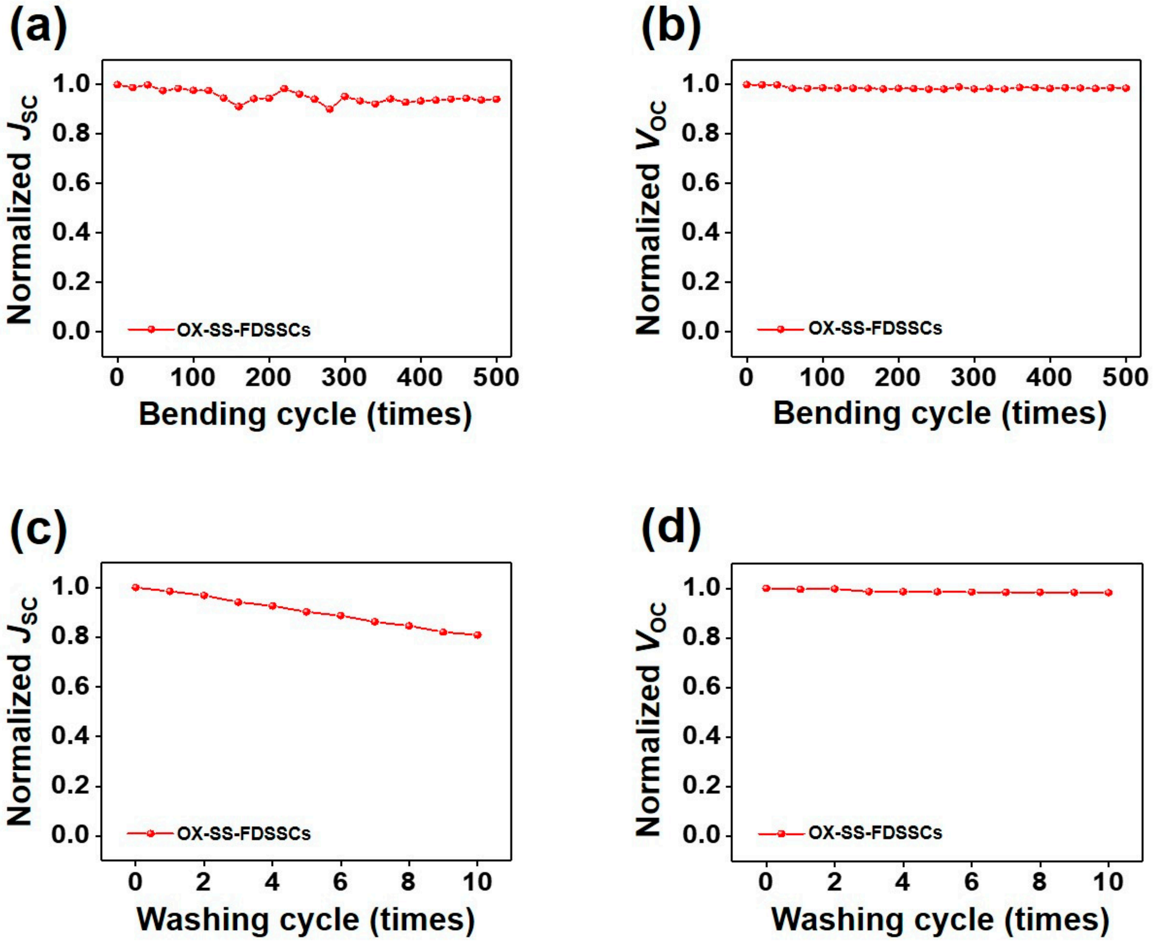

Figure S8. Normalized J_{SC} and V_{OC} of OX-enhanced SS-FDSSC as a function of (**a**) and (**b**) bending cycle, (**c**) and (**d**) washing cycle, respectively.

Table S1. EIS parameters of the SS-FDSSCs with TEMPO derivatives.

Device	R_s (Ω)	R_1 (Ω)	CPE$_1$ (F)	R_2 (Ω)	CPE$_2$ (F)
Pristine	25.0	41.5	3.36×10^{-6}	116	1.13×10^{-3}
OX	15.9	41.9	2.57×10^{-6}	104	1.38×10^{-3}
OAC	17.2	48.1	2.80×10^{-6}	144	9.44×10^{-4}
OBZ	19.9	39.3	2.87×10^{-6}	107	1.44×10^{-3}
